# Microcephaly and Chorioretinopathy Relevance as a Differential Diagnosis

**DOI:** 10.3390/diagnostics13152588

**Published:** 2023-08-03

**Authors:** Mauricio Bayram-Suverza, Karla Alejandra Torres-Navarro, Ángeles Yahel Hernández-Vázquez, Juan Abel Ramírez-Estudillo

**Affiliations:** Retina Department, Fundación Hospital de Nuestra Señora de La Luz, Mexico City 06030, Mexico

**Keywords:** chorioretinal atrophy, inherited diseases, microcephaly and chorioretinopathy, retina, retinal degeneration, genetics of retinal diseases

## Abstract

Microcephaly and chorioretinopathy are genetic disorders that are inherited in an autosomal recessive manner. The most frequent ocular manifestation is the presence of lacunar atrophy in the retina and choroid. The diagnosis of this condition can be challenging as several potential causes and related syndromes need to be ruled out. We present two cases of microcephaly and chorioretinopathy in Mexican patients, their clinical characterization, and discuss the differential diagnoses that should be considered. An 8-year-old girl was examined due to a history of decreased vision in both eyes. Fundus examination showed excavated, well-defined, sectorial, bilateral, and symmetrical areas of chorioretinal atrophy. An 18-year-old male had a history of poor vision since childhood. Previous ophthalmological examinations reported bilateral symmetric chorioretinal atrophy with pigment accumulation. Both patients had a prior diagnosis of microcephaly and language delay. Blood tests and a comprehensive systemic evaluation ruled out intrauterine infections. The electroretinogram showed decreased amplitude and increased implicit time in the photopic and scotopic responses. Genetic tests revealed mutations in the TUBGCP4 gene, leading to a diagnosis of microcephaly and chorioretinopathy. As observed in these cases, there was variability in retinal lesions. The presence of chorioretinal lacunae and genetic testing can help to correctly diagnose this disorder.

**Figure 1:**
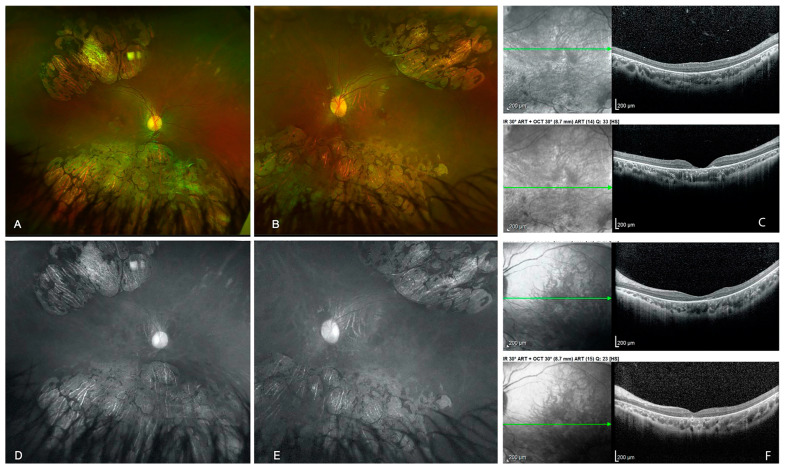
Ultra-wide field fundus photography of the right (**A**) and left (**B**) eyes, showing excavated, well-defined, sectorial, bilateral, and symmetrical chorioretinal atrophy. Foveal atrophy of the right eye observed using spectral domain-OCT (**C**). Red free photography of the right (**D**) and left (**E**) eyes with zones of chorioretinal atrophy. Less severe foveal atrophy in the left eye using spectral domain-OCT (**F**). Green arrows indicate the level at which the OCT scan was performed. Microcephaly is defined as a head circumference less than two standard deviations from the expected average for age, sex, and population. Its incidence varies from 1.3 to 150 cases per 100,000 births [1], and it can be diagnosed as a single disorder or as part of a syndrome secondary to congenital infections, hypoxic-ischemic damage, drug or alcohol exposure, metabolic alterations, or genetic mutations [2,3]. The most commonly reported ocular finding in genetic disorders associated with microcephaly is chorioretinal dysplasia, which in most series has been described as areas of lacunar atrophy or punch-out lesions that involve the retina and choroid and are considered non-progressive. This association has been observed in autosomal dominant and recessive inheritance patterns, in syndromes secondary to genetic mutations, and with significant expression variability. Other possible ocular findings are microphthalmia, myopic or hyperopic astigmatism, and Persistent Fetal Vasculature (PFV) [4,5]. Microcephaly and chorioretinopathy (MCCRP) is an autosomal recessive disorder characterized by dysmorphic facial features, microcephaly, developmental delay, and visual impairment with chorioretinopathy. The literature reports the association of this disorder with three genes: the tubulin-gamma complex-associated protein 6 (TUBGCP6) and 4 (TUBGCP4) genes and the polo-like kinase 4 (PLK4) gene. There is an autosomal dominant form of the disease associated with mutations of the kinesin family member 11 (KIF11) gene, called microcephaly with or without chorioretinopathy, lymphedema, or mental retardation (MCLMR), with several ocular and central nervous system alterations [2]. It has been suggested that some funduscopic findings may predict the underlying inheritance pattern. The focal areas of chorioretinal atrophy that resemble gyrate atrophy are more characteristic of the autosomal dominant form. In contrast, diffuse chorioretinal atrophy tends to occur in the autosomal recessive type associated with optic disc pallor, retinal vessel attenuation, and bone spicule formation [4,6]. Regarding systemic associations, the literature reports central nervous system abnormalities, psychomotor development delay, and heart defects, among others. Therefore, it is essential to perform a systemic approach when suspecting a diagnosis [6,7], highlighting the importance of ocular examination as exemplified by the two cases we report here. Sporadic microcephaly and chorioretinal dysplasia are diagnostic challenges requiring a multidisciplinary approach. It is necessary to perform a complete systemic evaluation focusing on cardiovascular, lymphatic, and central nervous system examinations. Magnetic resonance imaging is helpful since it usually reveals structural abnormalities that determine a cause and establish a prognosis [6]. As reported in the literature, our cases show that the visual spectrum ranges from normal to severely affected VA. The most frequent fundus findings are the areas of “lacunar” chorioretinal atrophy with a highly variable clinical presentation [4]. As previously highlighted, microcephaly with chorioretinopathy and microcephaly with familial exudative vitreoretinopathy have both been associated with mutations in KIF11, TUBGCP4, and TUBGCP6, and thus might represent a clinical spectrum rather than two distinct diagnoses. As explained, it is essential to keep this condition in mind as part of the differential diagnosis of pigmented atrophic lesions of the posterior pole in pediatrics. Conversely, it highlights the relevance of a complete ocular examination in patients with microcephaly. We present the cases of two Mexican patients with microcephaly who underwent an ophthalmological and systemic approach due to the presence of punched-out lesions in the fundus. After ruling out the associated systemic and ocular diseases, a genetic panel for hereditary retinal diseases confirmed the diagnosis of microcephaly and chorioretinopathy. The first case involves a female patient born prematurely at 36 weeks, the second child from nonconsanguineous healthy parents, diagnosed with microcephaly at four years old, who has been followed up by pediatric neurology ever since. She has a history of language delay. A previous external ocular evaluation reported probable retinitis pigmentosa at age 7. She was referred to our retina department at eight years old due to poor near vision. Ocular examination revealed a visual acuity (VA) of 20/80 and an intraocular pressure (IOP) of 12 mmHg in both eyes. Fundus examination revealed excavated, well-defined, sectorial, bilateral, symmetrical areas of chorioretinal atrophy and bull’s-eye maculopathy (Figure 1). We performed an electroretinogram (ERG) that showed decreased amplitude and increased implicit time in photopic and scotopic responses. Magnetic resonance imaging (MRI) showed no structural anomalies. Blood tests ruled out intrauterine infections (TORCH). A genetically inherited retinal disorder panel was used to rule out gyrate atrophy. However, a compound heterozygous pathogenic variant in the TUBGCP4 gene was detected (c.1678T > C (p.Trp560Arg)).

**Figure 2:**
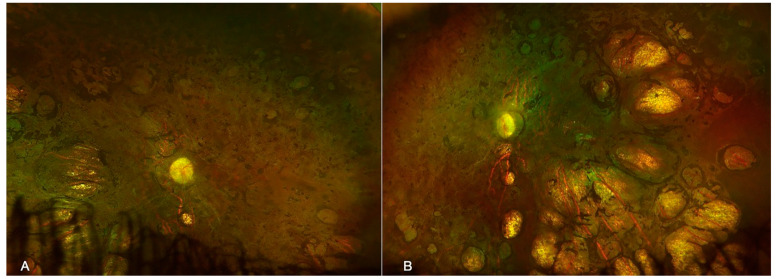
Ultra-wide field fundus photography of a male patient with microcephaly and chorioretinopathy of the right (**A**) and left (**B**) eyes showing excavated areas of chorioretinal atrophy and pigment accumulation, also known as “chorioretinal lacunae”. The second case was an 18-year-old male patient born at full term (first child), diagnosed with microcephaly at four months of age, with an MRI that revealed pachygyria and lissencephaly. In addition, he had a diagnosis of mild psychomotor retardation with a language deficit, and blood tests ruled out intrauterine infections. An ocular evaluation revealed a VA of 20/2000 and bilateral horizontal nystagmus in both eyes. Fundus examination revealed excavated areas of chorioretinal atrophy and pigment accumulation at a temporal location (Figure 2). An ERG showed subnormal and delayed photopic and scotopic responses. Genetic tests revealed compound heterozygous pathogenic variants in the TUBGCP4 gene. (c.1749G > T (silent) and TUBGCP4 c.1851 + 5G > A (intronic)).

## Data Availability

Not applicable.
